# Feelings of loneliness and meaning in life in subjects with Asperger’s syndrome: a pilot study

**DOI:** 10.1038/s41598-023-43826-z

**Published:** 2023-10-14

**Authors:** Kasper Sipowicz, Tadeusz Pietras, Marcin Kosmalski

**Affiliations:** 1 Department of Interdisciplinary Disability Studies, The Maria Grzegorzewska University in Warsaw, 02‐353 Warsaw, Poland; 2 The Second Department of Psychiatry, Institute of Psychiatry and Neurology in Warsaw, 02‐957 Warsaw, Poland; 3 Department of Clinical Pharmacology, Medical University of Lodz, 90‐153 Lodz, Poland

**Keywords:** Human behaviour, Psychology, Health care

## Abstract

Subjects with Asperger’s syndrome without intellectual disabilities have significant difficulties in establishing social relationships despite their IQ being within the normal range. One of the effects of social deficit is depression. The question arises whether loneliness and dimensions of meaning in life correlate with the severity of depression and whether the average severity of depression, loneliness and dimensions of meaning in life differentiate the following groups: people with Asperger’s syndrome and depression, people with Asperger’s syndrome without depression, people with depression without Asperger’s syndrome and healthy subjects. The study was conducted on a total of 170 people, including: 43 people with Asperger’s syndrome and depression, 41 people with Asperger’s syndrome without depression, 40 people with depression without Asperger’s syndrome and 46 healthy people (without Asperger’s syndrome and without depression). All were administered a demographic survey, Beck Depression Inventory II (BDI-II), De Jong Gierveld Loneliness Scale, Life Attitude Profile-Revised. Asperger’s syndrome and depressive episodes were diagnosed on the basis of ICD-10 research criteria still applicable in Poland. In the group with Asperger’s syndrome and depression the highest levels of loneliness and the lowest values of the dimensions of the sense of meaning in life, except for the acceptance of death, were observed. This result was significantly different from the results obtained in the other study groups. Both in people with Asperger’s syndrome without depression and in people with depression without Asperger’s syndrome, the values of the dimensions of the sense of meaning in life and the level of loneliness differ significantly from the results obtained in the control group. The BDI-II scores correlated positively with the loneliness values and negatively with the sense of meaning in life values in all groups. The results indicate that both suffering from depression and having Asperger’s syndrome are associated with an increased sense of loneliness and a reduced sense of meaning in life. People with Asperger’s syndrome and depression have the highest values of loneliness and the lowest values of dimensions of the sense of meaning of life compared to the other groups studied. The limitation of the work is the deliberate selection of groups, because it would be interesting to answer the question whether Asperger’s syndrome is a risk factor for depression in the population.

## Introduction

In the recent years, a paradigmatic reconstruction of the classification of mental disorders and behavioral disorders has been observed. In 2022/23, the World Health Organization (WHO) introduced the classification of mental and behavioral disorders contained in ICD-11, significantly different from the ICD-10 still in force in many countries^1–3^. In the USA, the DSM-5 classification was introduced in parallel^4,5^. People with Asperger’s syndrome according to ICD-10 are roughly people with autism spectrum without intellectual disability and without speech disorders^6–8^.

Recently, it has been noted that the difficulty of people with the ASD spectrum without intellectual disability in establishing contacts is associated with social isolation identified with loneliness and a subjective sense of loneliness and with a low sense of meaning in life^9–11^. The feeling of loneliness is understood as the subjective discrepancy between the expected and actual network of social support of the individual^12^. On the other hand, loneliness identified with social isolation is a sociometrically measurable current social support network^13^. The axial symptom of ASD is difficulty in establishing social relationships. The intellectual norm in this group of people gives them insight into their really small network of social support and can be the cause of a deep sense of loneliness^14,15^. This awareness may also be associated with the occurrence of phenomena such as a depressive episodes or anxiety disorders^16,17^. This insight into one’s own difficult situation may also be associated with a reduced sense of meaning in life^18^. This may be due to the fact that many interpersonal needs are not met due to difficult social contacts. The meaning of life according to Frankl is a state of subjective satisfaction of the individual resulting from purposeful and value-oriented actions^19,20^. In other words, it constitutes the essence and purpose of human existence, the vocation of man, which justifies the toil of life and makes it worth living^21–23^. The meaning in life can be considered both in its psychological and philosophical aspects. In the research on the relationship between the occurrence of illness and the meaning in life, this meaning is understood as a psychometrically measurable psychological construct^18^.

The aim of our work is to compare the sense of loneliness and the structure of the meaning in life in groups of people with Asperger’s syndrome and depression, with Asperger’s syndrome without depression, with depression without Asperger's syndrome and in a control group of healthy subjects. Our research aims to answer the question whether the co-morbidity of depression and Asperger’s syndrome has an additive effect on the feeling of loneliness and reduced sense of meaning in life in this group of patients.

It is also interesting whether the sense of meaning in life and the feeling of loneliness is more influenced by suffering from depression or being on the ASD spectrum without intellectual disability? Both disorders significantly interfere with the psychological functioning of a person and affect social contacts and purposeful activity of the subject.

In connection with the research objectives, the following research hypotheses were formulated:

1. The co-morbidity of depression and Asperger’s syndrome has an additive effect on the sense of loneliness and reduced sense of meaning in life of people affected by both these disorders.
2. The sense of meaning in life and the feeling of loneliness is affected more significantly by being on the ASD spectrum within the intellectual norm than by suffering from depression.

## Results

A group of 170 patients was included in the study, in which 43 patients were diagnosed with Asperger's syndrome and depression, 41 patients had Asperger's syndrome only, 40 patients had depression only, and 46 patients were healthy. The median age for all patients in the study was 27.00 (Q1–Q3: 22.00–33.00). Demographic data of patients included in the study are presented in Table 1.

**Table 1:** Demographics of patients included in the study.

Variable		Asperger syndrome and depression (N = 43)	Asperger syndrome (N = 41)	Depression (N = 40)	Healthy (N = 46)
Age^24^		25.00 (22.00–33.00)	25.00 (22.00–32.00)	27.50 (23.50–35.00)	28.00 (22.00–34.00)
Sex	Female	12 (27.91%)	3 (7.32%)	25 (62.50%)	24 (52.17%)
	Male	31 (72.09%)	38 (92.68%)	15 (37.50%)	22 (47.83%)
Marital status	Single	26 (60.47%)	36 (87.80%)	30 (75.00%)	16 (34.78%)
	In relation	17 (39.53%)	5 (12.20%)	10 (25.00%)	30 (65.22%)
Residence	With a parents	15 (34.88%)	27 (65.85%)	17 (42.50%)	10 (21.74%)
	With a partner	14 (32.56%)	5 (12.20%)	9 (22.50%)	28 (60.87%)
	Alone	14 (32.56%)	9 (21.95%)	14 (35.00%)	8 (17.39%)
Education	Secondary	36 (83.72%)	35 (85.37%)	22 (55.00%)	25 (54.35%)
	Professional	2 (4.65%)	0 (0.00%)	1 (2.50%)	2 (4.35%)
	Higher	5 (11.63%)	6 (14.63%)	17 (42.50%)	19 (41.30%)

Cronbach's alpha for the assessment of total internal consistency of all dimensions of the LAP-R scale was 0.77 (Table 2). Strong intercorrelations were observed between all dimensions of the scale, excluding Death Acceptance (DA) and 4 dimensions: Existential Vacuum (EV), Goal Seeking (GS), The Personal Meaning Index (TMPI) and Existential Transcendence (ET). The results are presented in Table S1 in the supplement.

**Table 2:** Internal consistency analysis for all dimensions of the LAP-R scale.

LAP-R	Median (Q1-Q3)	Mean ± SD	Cronbach’s Alpha	Correlation
Purpose (PU)	26.00 (22.00–33.00)	25.60 ± 8.86	0.70	0.98
Coherence (CO)	29.00 (24.00–34.00)	28.41 ± 7.22	0.71	0.96
Choice/responsibleness (CR)	33.00 (29.00–39.00)	31.46 ± 9.43	0.70	0.93
Death acceptance (DA)	32.00 (30.00–34.00)	31.74 ± 2.36	0.78	0.35
Existential vacuum (EV)	38.00 (31.00–43.00)	38.85 ± 9.82	0.90	-0.97
Goal seeking (GS)	33.00 (28.00–39.00)	32.34 ± 8.08	0.72	0.85
The personal meaning index (TPMI)	55.50 (47.00–67.00)	54.01 ± 15.82	0.64	0.98
Existential transcendence (ET)	47.00 (34.00–67.00)	46.02 ± 28.35	0.68	0.96
Total	–	–	0.77	–

*LAP-R* the Life Attitude Profile-Revised, *SD* standard deviation.

**Figure 1:**
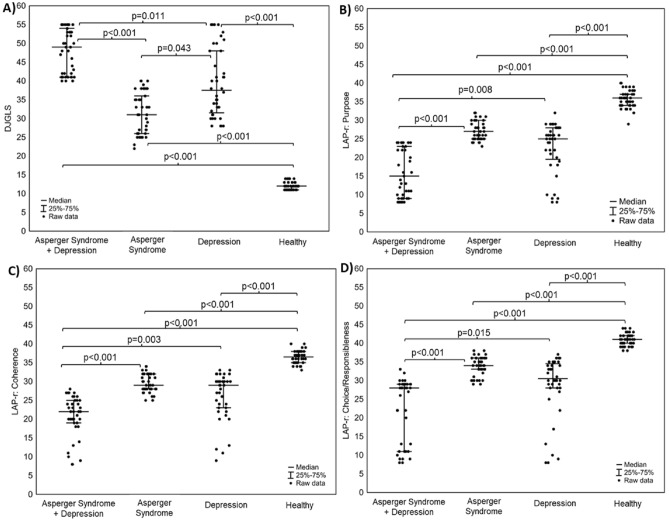

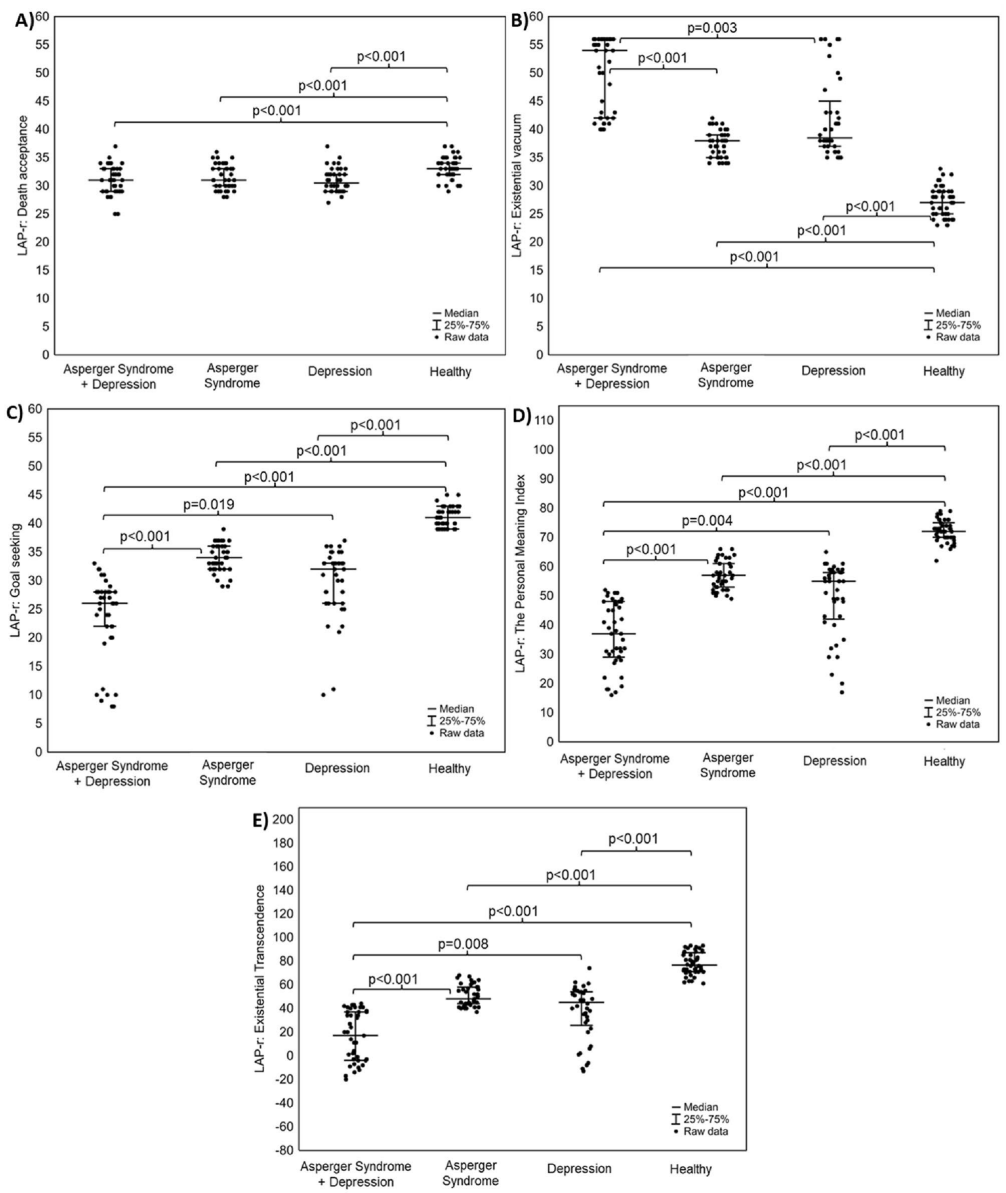
(**a**) Box-whisker charts of differences in the scores of loneliness rating scales (DJGLS scale) and sense of meaning in life (LAP-R scale) between 4 groups: patients with Asperger's Syndrome and depression, patients with Asperger's Syndrome only, patients with depression only, and healthy subjects; (**A**) feeling of loneliness (DJGLS scale); (**B**) Purpose (PU) on the LAP-R scale; (**C**) Coherence (CO) on the LAP-R scale, (**D**) Choice/Responsibleness (CR) on the LAP-R scale. (**b**) Box-whisker charts of differences in the scores of loneliness rating scales (DJGLS scale) and sense of meaning in life (LAP-R scale) between 4 groups: patients with Asperger’s. Syndrome and depression, patients with Asperger's Syndrome only, patients with depression only, and healthy subjects; **A**) Death Acceptance (DA) on the LAP-R scale **B**) Existential vacuum (EV) on the LAP-R scale; **C**) Goal-seeking (GS) on the LAP-R scale, D) The personal meaning index on the LAP-R scale, R) Existential transcendence (ET) on the LAP-R scale.

Statistically significant differences between patients with Asperger's syndrome and depression, patients with Asperger's syndrome only, patients with depression only, and healthy subjects were found in the values of the sense of loneliness in the DJGLS scale (Fig. 1a-A), as well as the values of dimensions in the LAP-R scale (Fig. 1a-B–D, b-A–E).

Patients with Asperger's syndrome and depression, compared to other groups, demonstrated the deepest sense of loneliness (Median (Q1–Q3): 49.00 (41.00–54.00)), the lowest sense of purpose (PU) (Median (Q1–Q3): 15.00 (9.00–54.00) 23.00)), the lowest scores in coherence (CO) (Median (Q1–Q3):22.00 (19.00–25.00)) and the lowest scores in the dimension of control of life in choice/responsibleness (CR) (Median (Q1-Q3):28.00 (11.00–25.00) 29.00)). In addition, patients with Asperger syndrome and depression obtained the highest scores in the existential vacuum (EV) dimension (Median (Q1–Q3): 54.00 (42.00–56.00)) and the lowest scores in the following dimensions: goal seeking (GS) (Median (Q1–Q3) : 26.00 (22.00–28.00)), the personal meaning index (TPMI) (Median (Q1–Q3): 37.00 (29.00–48.00)) and existential transcendence (ET) (Median (Q1–Q3): 17.00 (− 4.00–37.00)). There were no statistically significant differences between the ill patients in the three groups for the dimension of the LAP-R scale concerning the acceptance of death (DA). There was only a difference between the three groups of patients: Asperger's syndrome and depression, Asperger's syndrome, depression and the group of healthy subjects. Detailed results are presented in the supplement in Tables S2 and S3.

The analysis of the relationship between the DJGLS scale and the dimensions of the LAP-R scale and the severity of depression according to the Beck Depression Scale BDI-II, without division into groups (Table S4, Fig. S1, Supplement) showed strong, statistically significant correlations (*p* for all correlations < 0.001). A statistically significant, but weak, negative correlation was found only for death acceptance (DA) (R = − 0.395; *p* < 0.001). Group analysis, however, showed statistically significant correlations between all dimensions of the LAP-R and DJGLS scales and the results of depression severity in the BDI-II scale (Table 3).

**Table 3:** Correlations of Beck Depression Score (BDI-II) with DJGLS and LAP-R scale dimensions in 4 groups: patients with Asperger's syndrome and depression, patients with Asperger's syndrome, patients with depression and healthy subjects.

		Asperger syndrome and depression (N = 43)		Asperger syndrome (N = 41)		Depression (N = 40)		Healthy (N = 46)	
		R*	*p*	R*	*p*	R*	*p*	R*	*p*
DJGLS		0.889	< 0.001	0.938	< 0.001	0.921	< 0.001	0.817	< 0.001
LAP-R	PU	− 0.798	< 0.001	− 0.781	< 0.001	− 0.855	< 0.001	− 0.734	< 0.001
	CO	− 0.787	< 0.001	− 0.682	< 0.001	− 0.871	< 0.001	− 0.533	< 0.001
	CR	− 0.779	< 0.001	− 0.633	< 0.001	− 0.810	< 0.001	− 0.659	< 0.001
	DA	− 0.302	0.049	− 0.442	0.004	− 0.182	0.262	− 0.128	0.398
	EV	0.828	< 0.001	0.605	< 0.001	0.908	< 0.001	0.797	< 0.001
	GS	− 0.618	< 0.001	− 0.462	0.002	− 0.669	< 0.001	0.504	< 0.001
	TPMI	− 0.847	< 0.001	− 0.764	< 0.001	− 0.891	< 0.001	− 0.700	< 0.001
	ET	− 0.849	< 0.001	− 0.712	< 0.001	− 0.862	< 0.001	− 0.738	< 0.001

*R–R Spearman correlation coefficient.

The weakest correlation was found between death acceptance (DA) and severity of depression in the group with Asperger's syndrome and depression (weak, negative correlation: R = − 0.302; *p* = 0.049), as well as acceptance of death and severity of depression in the group with Asperger's syndrome (weak, negative correlation: R = − 0.442, *p* = 0.004). In all groups, the patients showed a strong increase in the sense of loneliness (DJGLS) with the increase in the severity of depression on the Beck scale (Asperger's syndrome and depression: R = 0.889, *p* < 0.001; Asperger's syndrome: R = 0.938, *p* < 0.001; Depression: R = 0.921, *p* < 0.001; 0.001; Healthy: R = 0.817, *p* < 0.001). In addition, patients with Asperger's Syndrome and depression had similar scores as patients in the depressed group for LAP-R dimensions such as: The personal meaning index (TPMI) (Asperger's Syndrome and depression: R = − 0.847, *p* < 0.001; Depression: R = -0.891, p < 0.001) and Existential transcendence (ET) (Asperger's syndrome and depression: R = − 0.849, *p* < 0.001; Depression: R = − 0.862, *p* < 0.001). Patients with Asperger's syndrome only in the case of TPMI and ET dimensions obtained a similar, average, negative correlation between the dimensions and the severity of depression on the Beck scale.

## Discussion

As it follows from our research, the patients with Asperger's syndrome and depression had the highest sense of loneliness, the lowest sense of purpose, the lowest sense of coherence, the lowest sense of control over life, and the highest values in existential vacuum. Both the autism spectrum and mood disorders have a dysfunctional effect on the broadly understood sense of self-efficacy. The autism spectrum is characterized by difficulties in establishing social relationships^25^. Establishing relationships is also hindered by the impaired functioning of the so-called theory of mind in people on the autism spectrum, as described by Baron Cohen^26^. The lack of the theory of mind makes it difficult to understand the intentions of another person, which results in difficulties in establishing relationships. On the other hand, withdrawal from social relations, anhedonia, and in extreme cases loss of the sense of meaning in life that may end in a suicide attempt are the features of a depressed mood^27^. However, it is not known whether the co-occurrence of Asperger's syndrome and depression is an independent product of events, or whether Asperger's syndrome promotes the development of depression^28^. Impaired ability to establish social contacts may, at least theoretically, contribute to the development of reactive mood disorders^29^. Exploration of the link between depression and autism is hampered by the lack of validated psychometric tools to identify mood problems in people on the autism spectrum^30^. The risk factors for mood disorders in people with autism are considered to largely overlap with those identified in the general population. However, they are probably exacerbated by the experience of autism and susceptibility to chronic stress associated with difficulties in social communication and sensory hypersensitivity typical of autism. Therefore, we assume that the studied group of people with Asperger's syndrome and depression may be more sensitive to rejection than people with Asperger's syndrome without depression. Hence, in the group of people with Asperger's syndrome and depression, we obtained lower values in terms of sense of meaning in life and low values within the range of scales measured by the LAP-R questionnaire. The distinction between depressive symptoms and autism spectrum symptoms is blurred by the recently discovered fact that the function of the mind is also disturbed in depression^31^.

Based on the analysis of works from the PubMed database, our publication is probably one of the first to address the problem of the sense of meaning in life in people with Asperger's syndrome. However, articles on the problem of loneliness in people with Asperger's syndrome have already been published^14^. One of these studies evaluated the relationship between friendship, loneliness and depressive symptoms in 35 people with Asperger's syndrome and 35 control subjects. People with Asperger's Syndrome reported poorer quality of best friendships and less motivation to develop them compared to the control group^14^. They were also characterized by a higher sense of loneliness and more severe depressive symptoms. The authors of the discussed work suggest that an increased level of negative affect may be associated with the poor quality of social relationships in the studied group of people. However, the authors do not propose a psychological model of the correlation between the observed variables. A 2015 review of studies preliminarily indicates that group interventions in well-functioning people on the autism spectrum reduce the feelings of loneliness and alleviate the co-occurrence of other psychopathological symptoms, including depressive symptoms^32^. Studies on the effectiveness of the UCLA PEERS program also demonstrated on a sample of 22 well-functioning adults with autism an improvement in social relationships and a reduction in feelings of loneliness in people participating in this program^33^.

In all four groups we studied, loneliness and all dimensions of the LAP-R scale, except the death acceptance dimension, correlated positively with the severity of depression (BDI-II). The coefficients of these correlations reached relatively high values at *p* < 0.001. The above means that the psychological construct of depression is similar to the psychological construct of the sense of loneliness and lack of meaning in life. It is puzzling that acceptance of death does not correlate with the results of the Beck Depression Inventory. This fact is explained by the well-known clinical observation that not every depressed patient has suicidal ruminations and acceptance of non-existence associated with the end of existence. A large proportion of patients with depression are afraid of death. Suicidal thoughts occur in severe depression, in exacerbations, and only patients in a stable state of mood disorders were qualified for our study. Our study should probably be repeated in a clinical ward where patients with suicidal ideations are hospitalized.

Due to the insufficient number of women representing the group with Asperger's syndrome and depression (N = 12), as well as with Asperger's syndrome (N = 3), statistical analysis could not be performed.

Due to the very similar age of all participants in the study, in all 4 groups, the results of the analysis of the correlation between the DJGLS scales and dimensions in the LAP-R scale and age were statistically insignificant, or statistically significant, but poorly correlated (Table S5 in the Supplement).

The results of the presented research provide important premises for the professional practice of psychiatrists and clinical psychologists. Special care should be given to people with co-occurrence of mood disorders and pervasive developmental disorders as a group particularly predisposed to a high sense of loneliness and a low sense of meaning in life.

## Limitations of the study

Too small group of people participating in the study prevented us from analyzing the studied variables in terms of gender and age, although Asperger's syndrome is believed to be several times more common in men than in women^34^. In the Polish population of people in the late adult phase, the estimated diagnosis of autism spectrum disorder is significantly underestimated, because the psychiatrists’ awareness of the existence of autism spectrum disorders appeared with the introduction of the ICD-10 classification in Poland. Seniors on the autism spectrum were usually classified as having intellectual disabilities. Therefore, our study involved only young adults diagnosed in childhood after the introduction of ICD-10 in Poland. What is missing from our paper is the reference to other research using psychometric tools such as DJGLS and LAP-R to study people on the autism spectrum and mood disorders. Such works either do not exist or are not available in English-language literature.

The selection of patients for individual groups was deliberate and was not random. It is interesting whether we would get similar results in large-population studies where patient selection would be random.

Another limitation of the study was the failure to include the duration of mood disorders in patients as a variable. The only analyzed variable was the severity of symptoms measured using the BDI-II scale. The use of antidepressants or the absence of their use were also not taken into account. However, based on the anamnesis it was known that most patients with depression took serotonin reuptake inhibitors or serotonin and norepinephrine reuptake inhibitors. In contrast, patients taking mood stabilizers had either a diagnosis of bipolar disorder or a history of hospitalization, which automatically excluded their participation in the study. Our goal was not to include the duration of the disease, or the method of treatment as a variable, but only the severity of symptoms measured quantitatively using a standardized tool—BDI-II.

## Conclusions

1. People with Asperger's syndrome and depression have higher values in terms of loneliness and lower values in terms of meaning in life than people with Asperger's syndrome without depression, people with depression without Asperger's syndrome, and healthy people.
2. The severity of depression measured with BDI-II strongly correlates with the intensity of loneliness and dimensions of meaning in life, except for the acceptance of death dimension.

## Methods

### Subjects

The study was carried out in the period 05.2022–03.2023 in the mental health clinic of the Society of Friends of the Disabled in Łódź and the family doctor’s clinic in Aleksandrów Łódzki. The study involved 170 people, i.e. 43 people with Asperger’s syndrome and depression, 41 with Asperger's syndrome without depression, 40 people with depression without Asperger’s syndrome and 46 healthy people. The patients were recruited from three mental health clinics, and the control group was selected from the charges of the family doctor. The selection of patients was deliberate. Efforts were made to ensure that the groups consisted of equal number of subjects, and people in each group were of a similar age. The study excluded people with mental and behavioral disorders other than a depressive episode and a high-functioning autism spectrum without intellectual disability. The study also excluded people hospitalized for psychiatric reasons within the last six months. People with severe somatic diseases, such as a past infarction or stroke in the last year, respiratory failure, COPD at risk level B, C, D, partially or poorly controlled bronchial asthma, heart failure grade III and IV according to NYHA, liver failure, chronic kidney disease in stages IIIa to Va according to KDIGO, thyrotoxicosis, active autoimmune diseases, cancer, inflammatory bowel disease, severe degenerative joint changes that make it difficult to move, decompensated type I and type II diabetes and other severe systemic diseases were also excluded. Therefore, 119 people were not enrolled in the study.

Subjects with depression and those with both depression and Asperger’s syndrome qualified for the study were diagnosed with F32 and F33 in a mild or moderate degree according to the ICD-10 classification still in force in Poland. In contrast, people with depression who had been hospitalized due to the severe course of the disease were excluded from the study because the severe course of depression and hospitalization could be an additional variable confounding the research results, both because of the stigma associated with the stay in psychiatric hospital and because of the lack of participation in social life caused by the symptoms of depression.

The patients completed the following questionnaires once: a demographic questionnaire of the authors’ own construction, the Beck’s Depression Inventory (BDI-II) in a version standardized and published by the Laboratory of Psychological Tests of the Polish Psychological Association, the Life Attitude Profile-Revised (LAP-R) by Gary T. Reker in a standardized version published by the Laboratory of Psychological Tests of the Polish Psychological Association, De Jong Gierveld Loneliness (DJGLS).

### Scales

The Polish version of De Jong Gierveld Loneliness Scale (DJGLS) adapted by Grygiel et al., with the consent of the author of the tool (Cronbach’s alpha 0.89) was used in the assessment of the feeling of loneliness^13,35,36^. The tool consists of eleven questions, with a five-point answer scale for each item. The increased total DJGLS score reflects a more severe feeling of loneliness^13^. A questionnaire was used to collect the demographic data such as the patients’ age, gender, marital status, residence and education level. To measure the sense of meaning in life, the Polish adaptation of the Life Attitude Profile-Revised (LAP-R) questionnaire (Cronbach's alpha between 0.70 and 0.80) was used^37,38^. The questionnaire, originally developed by Gary T. Reker, published by the Psychological Test Laboratory of the Polish Psychological Association, includes 8 scales. Six of them are simple scales, including Purpose (life goals and a sense of direction), Coherence (understanding oneself and the environment), Choice/Responsibleness (a view on the ability to make life choices), Death acceptance (no fear of death, accepting death as normal), Existential vacuum (absence of meaning in life, goals and direction), Goal seeking (desire for new experiences). Each item is rated from 1 (strongly disagree) to 7 (strongly agree) and each subscale has 8 items. The Existential vacuum scale is scored negatively, all the other scales positively. The calculation of the two remaining complex scales is based on the simple scales. They include The Personal Meaning Index (life goals, sense of direction, understanding of oneself and the environment), which is a sum of coherence and purpose, and Existential Transcendence (a general measure of life attitudes), which is a sum of purpose, coherence, choice/responsibleness, death acceptance with existential vacuum and goal seeking subtraction.

The severity of depressive symptoms (or depressiveness) was assessed with the use of the Beck Depression Inventory version II (BDI). The Polish version was adapted, validated and published by the Psychological Test Laboratory of the Polish Psychological Association. The test comprises 21 items concerning the occurrence and severity of depressive symptoms within the last two weeks. The scores for each item range from 0 to 3, which gives a total score of 0 to 63 points. The higher scores reflect the greater severity of depressiveness.

The study obtained the approval of the Bioethics Committee of the Medical University of Lodz (consent no. RNN/137/22/KE). Informed consent form was obtained from all participants in the study. All methods were performed in accordance with the relevant guidelines and regulations by including a statement in the methods section to this effect.

### Statistical analysis

In order to assess the internal consistency of the LAP-R scale in the study, Cronbach's alpha was used along with the assessment of intercorrelation between the dimensions of the scale. Nominal variables were presented using cardinality with percentages, while ordinal or continuous variables with a distribution other than normal were presented using medians with quartiles of 25 and 75% (Q1–Q3). The distribution of continuous variables was tested using the Shapiro–Wilk W test. The non-parametric Mann–Whitney U test was used to assess differences between the 2 groups, while non-parametric analysis of variance using the Kruskal–Wallis test was used to assess differences between > 2 groups. For statistically significant results, post-hoc tests were performed using Dunn's test. On the other hand, the non-parametric Spearman rank correlation test was used to assess the relationship between two continuous or ordinal variables, and the Spearman rank correlation coefficient (R) was used to assess the relationship between the variables. Statistically significant results for differences between two or more groups were presented using a box-whisker chart, and correlation results using a scatterplot. The level of statistical significance for the analyses was set at *p* < 0.05. The statistical program STATISTICA version 13.3 (TIBCO 2022, Poland) was used for the analyses.

### Supplementary Information

Supplementary Information.

## Data Availability

The datasets used and/or analyzed during the current study available from the corresponding author on reasonable request (marcin.kosmalski@umed.lodz.pl).
