# Supplementary material for: Feelings of loneliness and meaning in life in subjects with Asperger’s syndrome: a pilot study
Source: Sci Rep. 2023 Oct 14;13:17453. doi: 10.1038/s41598-023-43826-z (PMC10576817; doi:10.1038/s41598-023-43826-z)
Supplement: Supplementary file 1 — Supplementary Information. [file 41598_2023_43826_MOESM1_ESM.docx]

**Supplement**

**Table S1.** Intercorrelations between dimensions of the LAP-R scale

|  | **PU** | **CO** | **CR** | **DA** | **EV** | **GS** | **TMPI** | **ET** |
| --- | --- | --- | --- | --- | --- | --- | --- | --- |
| **PU** | 1.000 |  |  |  |  |  |  |  |
| **CO** | 0.934** | 1.000 |  |  |  |  |  |  |
| **CR** | 0.917** | 0.873** | 1.000 |  |  |  |  |  |
| **DA** | 0.321** | 0.320** | 0.288** | 1.000 |  |  |  |  |
| **EV** | -0.969** | -0.923** | -0.908** | -0.353** | 1.000 |  |  |  |
| **GS** | 0.856** | 0.873** | 0.840** | 0.245* | -0.846** | 1.000 |  |  |
| **TPMI** | 0.987** | 0.980** | 0.912** | 0.326** | -0.964** | 0.878** | 1.000 |  |
| **ET** | 0.974** | 0.935** | 0.941** | 0.413** | -0.975** | 0.798** | 0.972** | 1.000 |

**p<0.05 (two-sided)*

***p<0.001 (two-sided)*

**Table S2.** Differences in medians (Q1-Q3) between the 4 analyzed groups in the study

|  | | **Median**  **(Q1-Q3)** | | | |
| --- | --- | --- | --- | --- | --- |
|  |  | **Asperger Syndrome + Depression**  **(N=43)** | **Asperger Syndrome**  **(N=41)** | **Depression (N=40)** | **Healthy (N=46)** |
| **DJGLS** | | 49.00  (41.00 – 54.00) | 31.00  (26.00 – 36.00) | 37.50  (31.50 – 48.00) | 12.00  (11.00 – 12.00) |
| **LAP-R** | **PU** | 15.00  (9.00 – 23.00) | 27.00  (25.00 - 30.00) | 25.00  (19.50 - 28.00) | 36.00  (34.00 - 37.00) |
|  | **CO** | 22.00  (19.00 – 25.00) | 29.00  (28.00 – 32.00) | 29.00  (23.00 – 30.00) | 36.50  (35.00 – 38.00) |
|  | **CR** | 28.00  (11.00 – 29.00) | 34.00  (33.00 – 36.00) | 30.50  (28.00 – 34.50) | 41.00  (40.00 – 42.00) |
|  | **DA** | 31.00  (29.00 – 33.00) | 31.00  (30.00 – 33.00) | 30.50  (29.00 – 32.00) | 33.00  (32.00 – 34.00) |
|  | **EV** | 54.00  (42.00 – 56.00) | 38.00  (35.00 – 39.00) | 38.50  (37.00 – 45.00) | 27.00  (25.00 – 29.00) |
|  | **GS** | 26.00  (22.00 – 28.00) | 34.00  (32.00 – 36.00) | 32.00  (26.00 – 33.00) | 41.00  (39.00 – 43.00) |
|  | **TPMI** | 37.00  (29.00 – 48.00) | 57.00  (53.00 – 61.00) | 55.00  (42.00 – 58.00) | 72.00  (70.00 – 75.00) |
|  | **ET** | 17.00  (-4.00 – 37.00) | 48.00  (44.00 – 58.00) | 44.00  (25.50 – 54.00) | 76.50  (71.00 – 87.00) |

**Table S3.** Results of post-hoc statistical significance of Dunn's test analysis for comparison of the Loneliness Scale (DJGLS) and LAP-R scale dimensions in the compared 4 groups

| **DJGLS vs 4 groups** | | | |
| --- | --- | --- | --- |
|  | **Asperger Syndrome + Depression** | **Asperger Syndrome** | **Depression** |
| **Asperger Syndrome** | **<0.001** | **-** | **-** |
| **Depression** | **0.011** | **0.043** | - |
| **Healthy** | **<0.001** | **<0.001** | **<0.001** |
| **LAP-R: Purpose** | | | |
|  | **Asperger Syndrome + Depression** | **Asperger Syndrome** | **Depression** |
| **Asperger Syndrome** | **<0.001** | **-** | **-** |
| **Depression** | **0.008** | 0.114 | - |
| **Healthy** | **<0.001** | **<0.001** | **<0.001** |
| **LAP-R: Coherence** | | | |
|  | **Asperger Syndrome + Depression** | **Asperger Syndrome** | **Depression** |
| **Asperger Syndrome** | **<0.001** | **-** | **-** |
| **Depression** | **0.003** | 0.460 | - |
| **Healthy** | **<0.001** | **<0.001** | **<0.001** |
| **LAP-R: Choice/Responsibleness** | | | |
|  | **Asperger Syndrome + Depression** | **Asperger Syndrome** | **Depression** |
| **Asperger Syndrome** | **<0.001** | **-** | **-** |
| **Depression** | **0.015** | 0.089 | - |
| **Healthy** | **<0.001** | **<0.001** | **<0.001** |
| **LAP-R: Death Acceptance** | | | |
|  | **Asperger Syndrome + Depression** | **Asperger Syndrome** | **Depression** |
| **Asperger Syndrome** | >0.999 | **-** | **-** |
| **Depression** | >0.999 | >0.999 | - |
| **Healthy** | **0.001** | **0.011** | **<0.001** |
| **LAP-R: Existential vacuum** | | | |
|  | **Asperger Syndrome + Depression** | **Asperger Syndrome** | **Depression** |
| **Asperger Syndrome** | **<0.001** | **-** | **-** |
| **Depression** | **0.003** | 0.315 | - |
| **Healthy** | **<0.001** | **<0.001** | **<0.001** |
| **LAP-R: Goal Seeking** | | | |
|  | **Asperger Syndrome + Depression** | **Asperger Syndrome** | **Depression** |
| **Asperger Syndrome** | **<0.001** | **-** | **-** |
| **Depression** | **0.019** | 0.054 | - |
| **Healthy** | **<0.001** | **<0.001** | **<0.001** |
| **LAP-R: The Personal Meaning Index** | | | |
|  | **Asperger Syndrome + Depression** | **Asperger Syndrome** | **Depression** |
| **Asperger Syndrome** | **<0.001** | **-** | **-** |
| **Depression** | **0.004** | 0.218 | - |
| **Healthy** | **<0.001** | **<0.001** | **<0.001** |
| **LAP-R: Existential Transcendence** | | | |
|  | **Asperger Syndrome + Depression** | **Asperger Syndrome** | **Depression** |
| **Asperger Syndrome** | **<0.001** | **-** | **-** |
| **Depression** | **0.008** | 0.254 | - |
| **Healthy** | **<0.001** | **<0.001** | **<0.001** |

**Table S4.** Correlation of the severity of depression in the Beck Scale (BDI-II) and the Loneliness Scale (DJGLS) and dimensions in the LAP-R scale for all patients without grouping (N=170)

|  |  | **LAP-R** | | | | | | | |
| --- | --- | --- | --- | --- | --- | --- | --- | --- | --- |
|  | **DJGLS** | **PU** | **CO** | **CR** | **DA** | **EV** | **GS** | **TMPI** | **ET** |
| **BDI-II** | 0.954* | -0.932* | -0.912* | -0.920* | -0.395* | 0.923* | -0.868* | -0.930* | -0.923* |

**p<0.001 (two-sided)*


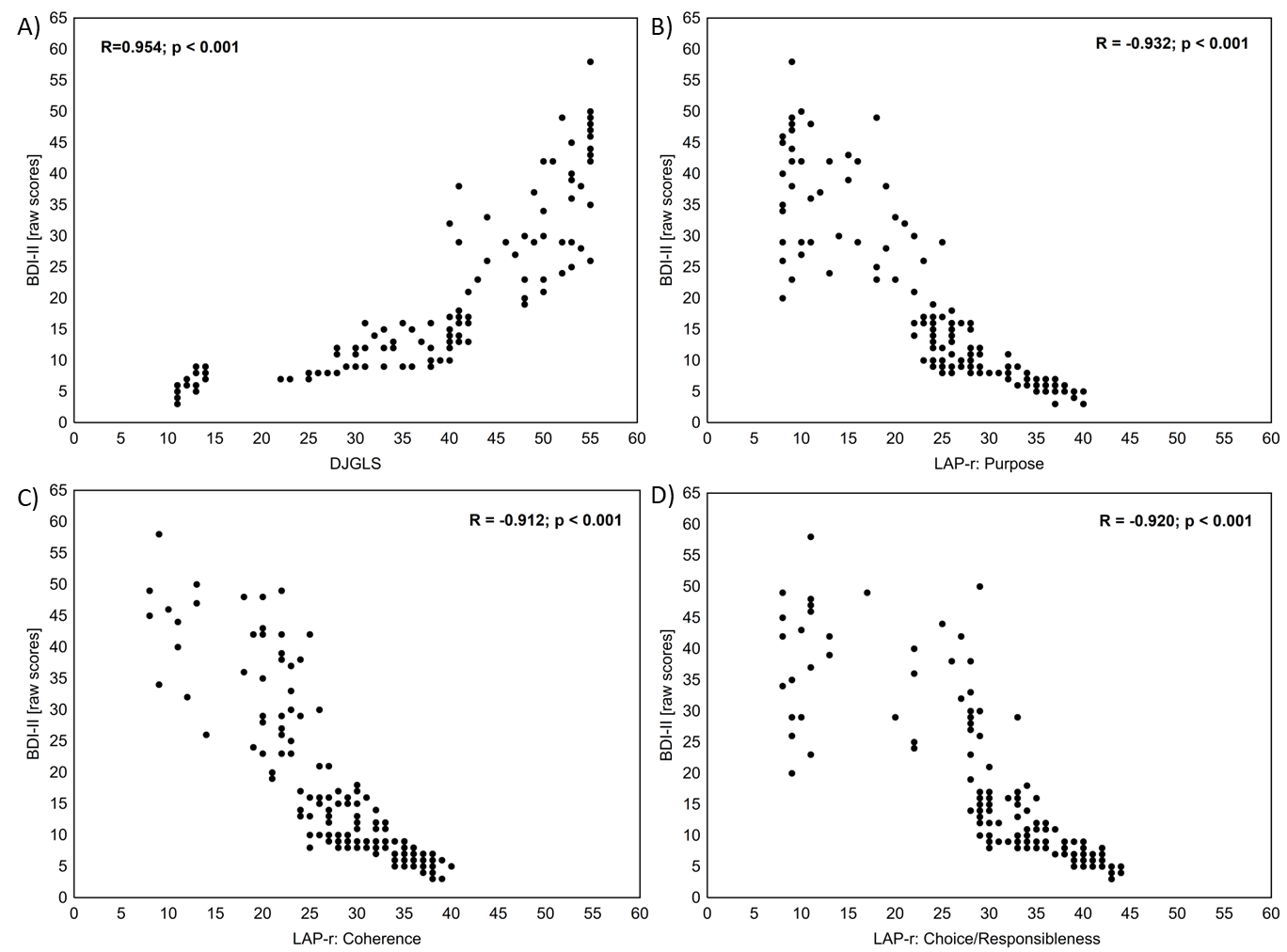


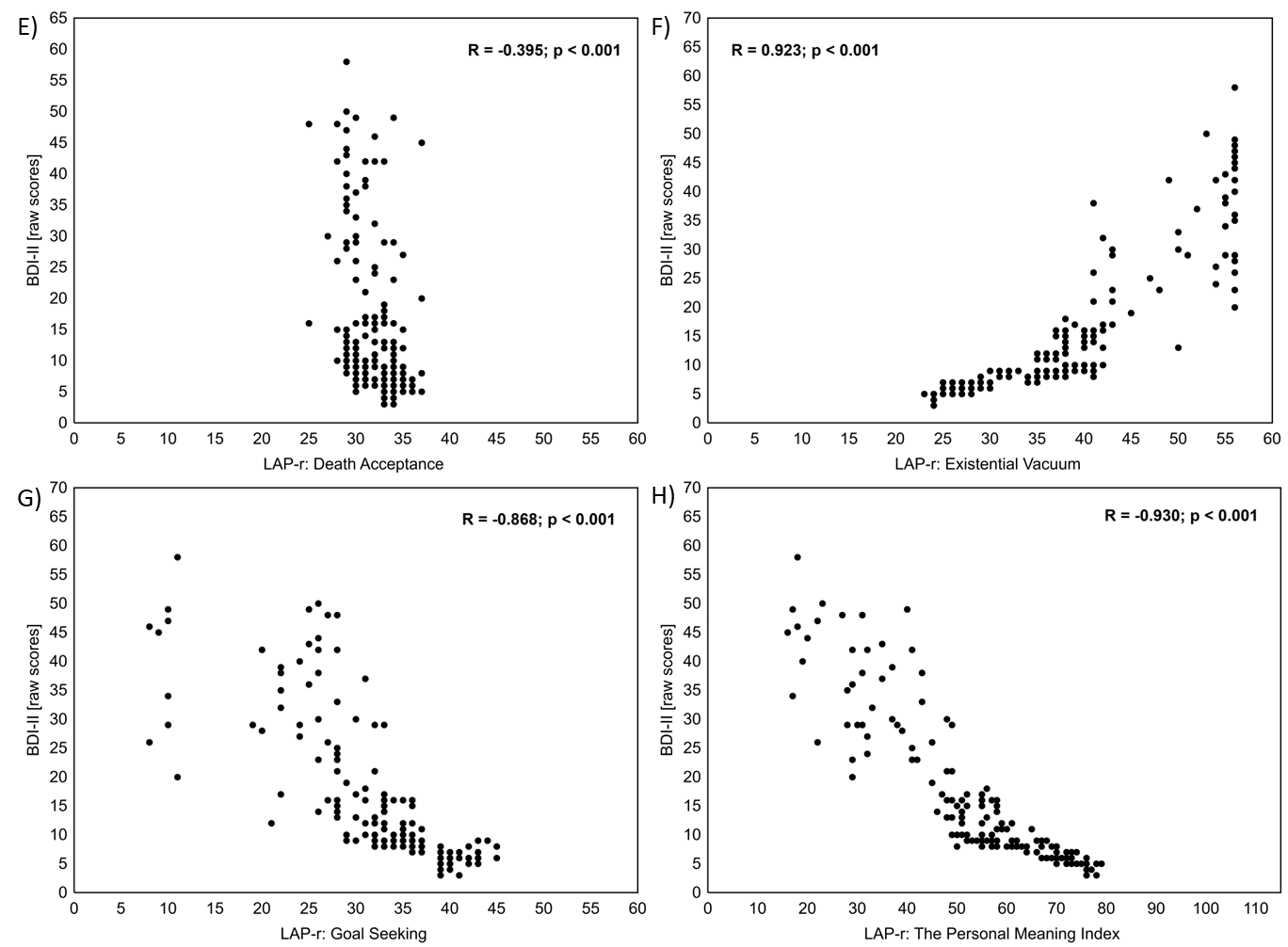


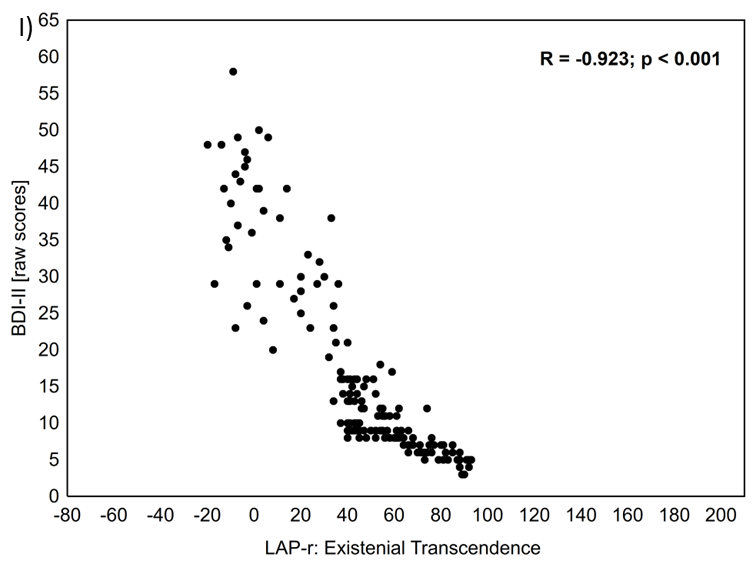


**Figure S1.** Scatterplots of correlation between the severity of depression in the Beck scale (BDI-II) and the feeling of loneliness in the DJGLS scale and dimensions in the LAP-R scale for all patients without division into groups

**Table S5.** Correlation of age with the DJGLS scale and LAP-R scale dimensions divided into 4 study groups

|  | | **Age [Years]** | | | | | | | |
| --- | --- | --- | --- | --- | --- | --- | --- | --- | --- |
|  |  | **Asperger syndrome and depression (N=43)** | | **Asperger syndrome (N=41)** | | **Depression (N=40)** | | **Healthy (N=46)** | |
|  |  | **R*** | **p** | **R*** | **p** | **R*** | **p** | **R*** | **p** |
| **DJGLS** | | -0.252 | 0.103 | -0.163 | 0.308 | -0.238 | 0.139 | -0.211 | 0.159 |
| **LAP-R** | **PU** | 0.200 | 0.199 | 0.313 | **0.047** | 0.308 | 0.053 | 0.484 | **<0.001** |
|  | **CO** | 0.259 | 0.093 | 0.467 | **0.002** | 0.349 | **0.027** | 0.539 | **<0.001** |
|  | **CR** | 0.381 | **0.012** | 0.145 | 0.367 | 0.280 | 0.080 | 0.120 | 0.428 |
|  | **DA** | -0.001 | 0.993 | -0.040 | 0.804 | -0.326 | **0.040** | -0.035 | 0.817 |
|  | **EV** | -0.334 | **0.029** | -0.465 | **0.002** | -0.300 | 0.060 | -0.298 | **0.045** |
|  | **GS** | -0.032 | 0.837 | -0.114 | 0.477 | 0.062 | 0.702 | -0.590 | **<0.001** |
|  | **TPMI** | 0.219 | 0.159 | 0.406 | **0.008** | 0.364 | **0.021** | 0.561 | **<0.001** |
|  | **ET** | 0.350 | **0.021** | 0.425 | **0.006** | 0.287 | 0.072 | 0.433 | **0.003** |
